# The Surgery of the Vermiform Appendix

**Published:** 1904-07-23

**Authors:** 


### THE SURGERY OF THE VERMIFORM APPENDIX.

Appendicitis.—Cotterill[1] says that pain increasing after the first 36 hours points to the formation of pus or to commencing gangrene of the appendix. If the pain slowly decreases it points to a subsidence of the inflammation; if, on the other hand, the pain quickly disappears, the case must be regarded with grave suspicion, as it frequently indicates gangrene. Tenderness may only be elicited on a digital examination by the rectum or vagina. Rigidity of the lower right quadrant does not appear at once, but only when pain is felt over the appendix, and this rigidity is progressive. The temperature should begin to drop in the morning after the second day. A sudden drop in the temperature often indicates perforation, but the condition of the pulse is a guide in such an event. Such symptoms as persistent vomiting, quick and weak pulse out of proportion to the temperature, abdominal facies, rigors, and tympanites point not to the threatening of a bad attack, but to the fact that the patient has already had a severe blow from the effects of which nothing might save him. He summarises the indications for surgical interference as follows:—In fulminating or perforating cases immediate operation is the only successful means of treatment. In other less acute cases the following points indicate operation:—1. Vomiting continued after 12 hours. 2. A pulse-rate of a hundred and upwards, and out of proportion to the temperature. 3. A temperature suddenly falling or rising after the second day, especially if there are marked remissions. 4. Pain suddenly stopping or, after the second day, markedly rising An acute onset of symptoms with rapid pulse, continued vomiting, and tenderness over the appendix without a tumour in the right iliac fossa should call for immediate operation. On the other hand, a quiet onset with a pulse-rate under a hundred, and early formation of tumour in the right iliac fossa might allow of a safe delay, but the surgeon should operate immediately on the first appearance of bad symptoms. Dean[2] considers that when once appendicitis is diagnosed there is only one proposition to be made, namely, to urge operation at the earliest possible moment, for no one is able to prognosticate the course of the disease in the beginning of the first attack. Baldy[3] holds that appendicitis does not generate pelvic inflammatory disease, nor does pelvic inflammatory disease cause appendicitis. Ninety-five per cent. of the mixed cases are in origin cases of pelvic inflammatory disease, and the adhesion of the vermiform appendix is a mere coincidence due to inflammation of contiguous serous surfaces, as was so commonly the case of adhesion of intestines in pelvic inflammatory diseases. He has never seen a single case in which, having found pus in a Fallopian tube, there was also pus in the involved vermiform appendix; nor has he found a perforated or gangrenous appendix in such a case. Barnard[5] thinks that anybody who has had a first attack of appendicitis should have his or her appendix removed. In such cases the mortality is only $\frac{1}{3}$ per cent. when done by a competent surgeon. By having the appendix removed the patient will avoid the risk of subsequent attacks with an average mortality of 5 per cent. at each attack. He is opposed to appendicectomy during the second, third, or fourth day, except as a forlorn hope, or when rendered imperative by the case becoming progressively worse, because to find the appendix it would be necessary to break down the lymph-barriers which are shutting off the general peritoneal cavity from the acute process going on. Cheatle,[5] however, does not believe that after the evacuation of pus any harm is done by breaking through the remaining walls in looking for and removing an accessible appendix. Berry[6] thinks that if a large incision is made in the abdominal wall, one or more nerves are cut, an accident which leads to paralysis and atrophy of the muscular fibres, and so favours the occurrence of ventral hernia. Referring to the removal of the appendix during an acute attack, he thinks that the risks of the operation are far more serious when performed at that period of the disease. During the acute attack the septic focus is not limited to the interior of the appendix, as it is in the intervening period; and, therefore, appendicectomy in the acute stage undoubtedly involves risk of disseminating that septic matter very widely. Curschmann[7] considers the relationship of leucocytosis and appendicitis and concludes as follows:—That in non-suppurative cases there is either no increase of leucocytes or only a moderate leucocytosis which disappears as the inflammation subsides. A leucocytosis of as much as 22,000 may be present for a few days only at the commencement of an acute attack without subsequent suppuration. A leucocyte count of 25,000 or over on a single occasion is presumptive evidence, and if it continues, positive evidence of the occurrence of suppuration. Wassermann[8] insists that the leucocyte count must only be looked upon as one clinical factor to be depended upon in coming to a conclusion as to the presence or absence of suppuration. Interesting cases are recorded by Footner[9] and Barling.[10]

[1] Lancet, Jan. 30, 1904, p. 301. [2] Med. Rec., Jan. 9, 1904, p. 73. [3] Ibid. [4] The Clin. Jour., Feb. 10, 1904, p. 269. [5] Ibid., p. 271 [6] The Clin. Jour., Feb. 17, 1904, p. 282. [7] Med. Chron., Jan. 1904. p. 257. [8] Ibid. [9] Brit. Med. Jour. Dec. 26, 1903, p. 1640. [10] Birmingham Med. Rev., Feb. 1904, p. 61.

